# What is the impact of human umbilical cord mesenchymal stem cell transplantation on clinical treatment?

**DOI:** 10.1186/s13287-020-02011-z

**Published:** 2020-12-01

**Authors:** Qixin Xie, Rui Liu, Jia Jiang, Jing Peng, Chunyan Yang, Wen Zhang, Sheng Wang, Jing Song

**Affiliations:** 1 Anhui Key Laboratory, Department of Pharmacy, Yijishan Hospital Affiliated to Wannan Medical College, Wuhu, China; 2 Department of Medical Laboratory, Yijishan Hospital of Wannan Medical College, Wuhu, China

**Keywords:** Human umbilical cord mesenchymal stem cells, Clinical application, Therapy

## Abstract

**Background:**

Human umbilical cord mesenchymal stem cells (HUC-MSCs) present in the umbilical cord tissue are self-renewing and multipotent. They can renew themselves continuously and, under certain conditions, differentiate into one or more cell types constituting human tissues and organs. HUC-MSCs differentiate, among others, into osteoblasts, chondrocytes, and adipocytes and have the ability to secrete cytokines. The possibility of noninvasive harvesting and low immunogenicity of HUC-MSCs give them a unique advantage in clinical applications. In recent years, HUC-MSCs have been widely used in clinical practice, and some progress has been made in their use for therapeutic purposes.

**Main body:**

This article describes two aspects of the clinical therapeutic effects of HUC-MSCs. On the one hand, it explains the benefits and mechanisms of HUC-MSC treatment in various diseases. On the other hand, it summarizes the results of basic research on HUC-MSCs related to clinical applications. The first part of this review highlights several functions of HUC-MSCs that are critical for their therapeutic properties: differentiation into terminal cells, immune regulation, paracrine effects, anti-inflammatory effects, anti-fibrotic effects, and regulating non-coding RNA. These characteristics of HUC-MSCs are discussed in the context of diabetes and its complications, liver disease, systemic lupus erythematosus, arthritis, brain injury and cerebrovascular diseases, heart diseases, spinal cord injury, respiratory diseases, viral infections, and other diseases. The second part emphasizes the need to establish an HUC-MSC cell bank, discusses tumorigenicity of HUC-MSCs and the characteristics of different in vitro generations of these cells in the treatment of diseases, and provides technical and theoretical support for the clinical applications of HUC-MSCs.

**Conclusion:**

HUC-MSCs can treat a variety of diseases clinically and have achieved good therapeutic effects, and the development of HUC-MSC assistive technology has laid the foundation for its clinical application.

## Introduction

HUC-MSCs are self-renewing and multipotent. They can continuously proliferate and differentiate under specific conditions into one or more cell types that constitute human tissues and organs. They affect immune responses and can be easily harvested, separated, cultured, expanded, and purified. HUC-MSCs retain the stemness after multiple passages and expansion. The surface antigens of HUC-MSCs are not prominent, the rejection of transplanted cells is insignificant, and the matching requirements are not strict, facilitating their use in allografts [1–3].

At present, HUC-MSCs are used in the treatment of various diseases. They have several distinct properties essential for their therapeutic applications. (1) Differentiation: the generation of differentiated cells by HUC-MSCs promotes tissue regeneration and improves tissue function [4, 5]. (2) Immune regulation: HUC-MSCs inhibit the proliferation of immune cells, such as T cells, B cells, and Tfh cells; induce the differentiation of macrophages from pro-inflammatory phenotypes to anti-inflammatory phenotypes; and reduce inflammation by secreting interleukin-10 (IL-10) and interleukin-4 (IL-4). Together, these modifications of immune responses facilitate tissue repair [5]. (3) Paracrine effects: HUC-MSCs promote tissue regeneration by secreting soluble molecules such as keratinocyte growth factor (KGF), hepatocyte growth factor (HGF), epidermal growth factor (EGF), and other cytokines [5–7]. (4) Anti-inflammatory effect: HUC-MSCs suppress the secretion of inflammatory factor interleukin-1β (IL-1β), tumor necrosis factor-α (TNF-α), and interleukin-8 (IL-8), reducing inflammation and oxidative stress, thus suppressing cell apoptosis [8, 9]. (5) Anti-fibrotic activity: HUC-MSCs stimulate fibrosis-related cell apoptosis and the secretion of HGF and other molecules. The anti-fibrotic function can also be mediated by the regulation of related signaling pathways and the promotion of vascular remodeling. (6) Non-coding RNA regulation: HUC-MSCs can affect the expression of microRNA (miRNA), long non-coding (lncRNA), and circular RNA (circRNA), indirectly regulating their target genes and achieving therapeutic effects [10–12].

**Fig. 1:**
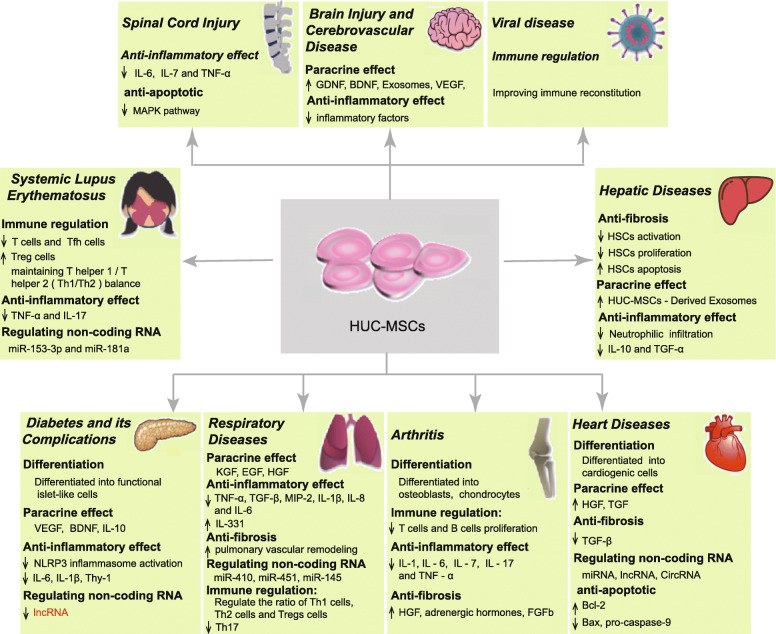
Clinical application and mechanisms of action of HUC-MSCs. This figure depicts the use of HUC-MSCs in the treatment of various diseases such as diabetes and its complications, liver diseases, systemic lupus erythematosus, arthritis, brain injury and cerebrovascular disease, heart diseases, spinal cord injury, respiratory diseases, viral infections, and other diseases

Currently, HUC-MSCs are used to treat more than ten types of diseases, and major therapeutic breakthroughs have been achieved with these cells. In this review, we will summarize the progress in the application of HUC-MSCs during the last 5 years, with the objective of guiding further research and clinical applications (Fig. 1, Table S1).

## Main text

### Application of HUC-MSCs in clinical treatment

#### Application of HUC-MSCs in diabetes and its complications

Diabetes is a group of metabolic diseases characterized by hyperglycemia resulting from insufficient insulin secretion, impaired response to insulin, or both [13]. Clinically, two types of diabetes are recognized: type 1 insulin-dependent diabetes mellitus (T1DM) and type 2 insulin-independent diabetes mellitus (T2DM). T1DM is typically characterized by a low level of insulin and C-peptide due to the impairment of islet β cell function, while T2DM is associated with the reduction in insulin receptor sensitivity. It has been shown that HUC-MSCs injected intravenously in diabetic animals can home to pancreatic islets and differentiate into functional islet-like cells. These cells affect macrophage polarization while blocking the activation of NLRP3 inflammasome and inflammatory factors [8, 14]. These anti-inflammatory effects improve the course of diabetes. In diabetic patients, 6 months to 1 year after intravenous injection of HUC-MSCs, the metabolic index was improved, the level of insulin and C-peptide was increased, the number of Treg cells was elevated, while glycosylated hemoglobin, fasting glucose, and daily insulin requirement were decreased [15, 16]. HUC-MSCs are safe and effective in the treatment of diabetes.

Diabetic complications, such as diabetic foot, diabetic nephropathy, diabetic wound ulcers, and diabetic retinopathy, are frequently the primary causes of disability and death. In the clinical treatment of diabetic foot, HUC-MSCs could be targeted to the ulcer and increase the formation of vascular endothelial growth factor (VEGF) and brain-derived neurotrophic factor (BDNF). These factors could promote the epithelialization of ulcerated tissue by stimulating the release of cytokeratin 19 from keratinocytes and extracellular matrix formation [17–19]. All treated patients exhibited significant improvement in ankle-brachial pressure index, transcutaneous oxygen tension, and claudication distance. Moreover, the density of newly formed vessels increased, and ulcers healed partially or completely [20]. Based on these results, HUC-MSCs were suggested to provide an effective strategy for the treatment of the diabetic foot.

When HUC-MSCs have been applied in the treatment of diabetic nephropathy, they exerted a therapeutic effect by reducing the expression of inflammatory cytokines, increasing the number of Sertoli cells, and upregulating the expression of their proteins and enhancing the expression of anti-apoptotic proteins in the kidney [21]. Experiments in diabetic rats documented that blood glucose, blood urea nitrogen, and 24-h urinary albumin excretion rate were significantly reduced after the treatment [22]. In a clinical study, 5 patients aged 30–60 years, with chronic diabetic non-healing wounds, received HUC-MSC transplantation and were followed up for 1 month. The healing time and the size of the wound significantly shorten after HUC-MSC treatment [23], but the mechanism responsible for this benefit was not clear. It may be related to macrophage polarization; increased secretion of IL-10, VEGF, and other cytokines; decreased secretion of IL-6; or upregulation of certain genes [24, 25]. In animal studies on the treatment of diabetic retinopathy, HUC-MSCs were induced to differentiate into neurological function cells in vitro and then transplanted in vivo. With time, retinal microvascular permeability and vessel leakage were reduced. Moreover, the expression of Thy-1, IL-1β, IL-6, lncRNA, and myocardial infarction-related transcript (MIAT) was significantly reduced [26, 27], indicating that HUC-MSCs represent a promising candidate for application in the treatment of diabetic retinopathy.

In summary, HUC-MSCs have the following characteristics: (1) targeting inflammatory tissues and differentiation into functional islet-like cells to block the activity of inflammasomes and exert anti-inflammatory effects; (2) targeting the ulcer tissue and promote the release of cytokines, and then promote the epithelialization of ulcerated tissue, eventually resulting in partial or complete healing of the ulcer; and (3) potential to differentiate in vitro into functional cells and being transplanted in vivo, providing a satisfactory therapeutic effect. Therefore, we believe that when HUC-MSCs are used therapeutically, they need first to target damaged or inflamed tissues, and then differentiate into differentiated cells or secrete immune regulatory factors. HUC-MSCs can also be induced in vitro to differentiate into functional cells and subsequently be used for transplantation to produce adequate therapeutic outcomes.

#### Application of HUC-MSCs in hepatic diseases

Hepatitis, cirrhosis, and liver cancer are common liver diseases, and fibrosis is the common pathway underlying the development of multiple chronic conditions of the liver. The activation of hepatic stellate cells (HSCs) is a critical element of the etiology of hepatic fibrosis [28], which can be inhibited by HUC-MSCs. HUC-MSCs suppress the proliferation of HSCs by downregulating the expression of transforming growth factor-1 (TGF-1) and Smad3 while increasing the expression of Smad7 [29]. Studies in animal models demonstrated that HUC-MSCs accelerate the degradation of fiber matrix and promote the apoptosis of HSCs by increasing the expression of matrix metalloproteinases (MMPs), particularly MMP-13 [30]. HUC-MSCs co-cultured with activated HSCs inhibited their proliferation and induced cell apoptosis by reducing collagen deposition [29, 30]. Moreover, HUC-MSCs can prevent the activation of HSCs via paracrine mechanisms, blocking the synthesis of IL-10 and TGF-α and, thereby, eliminating the inhibitory effect of HUC-MSCs on HSC proliferation and collagen production [31]. Thus, HUC-MSCs appear as an important regulator of HSC proliferation and apoptosis, implying that the infusion of HUC-MSCs can delay or even reverse liver fibrosis and consequent liver diseases. Besides, HUC-MSC-derived exosomes (HUC-MSC-ex) could reduce the expression of the NLRP3 inflammasome by inhibiting the activation of proteins associated with this complex and decreasing the level of alanine transaminase (ALT), aspartate aminotransferase (AST), and pro-inflammatory cytokines, playing an anti-inflammatory role [32]. At the same time, HUC-MSCs-ex could also reduce the infiltration of neutrophils, and oxidative stress and apoptosis of liver cells in vivo, and function as an antioxidant protecting the liver against oxidative damage and ischemia-reperfusion injury [9]. The transplantation of HUC-MSCs had obvious hepatoprotective effects as it significantly improved hepatocellular necrosis and neutrophilic infiltration without triggering serious adverse reactions or tumor formation [33]. Therefore, HUC-MSCs may provide new treatment strategies for liver fibrosis and other liver diseases.

We believe that HUC-MSCs could treat liver diseases based on the following properties: (1) HUC-MSCs inhibit proliferation and promote apoptosis of HSCs, delaying or even reversing liver fibrosis and fibrosis-related liver diseases; (2) HUC-MSCs release exosomes that can reduce the expression of NLRP3 inflammasomes and decrease the level of pro-inflammatory factors, thereby achieving an anti-inflammatory effect; and (3) HUC-MSCs can reduce the level of ALT and AST, suppress the infiltration by neutrophils, decline oxidative stress and apoptosis of liver cells, and protect against oxidative and ischemia-reperfusion injury of the liver. At present, initial steps in the field of the application of HUC-MSCs for the treatment of liver diseases have been made, but in-depth research on the underlying mechanism of action remains to be performed. For example, the optimal time for transplantation, the administration method, and the effective dosage need to be determined. In addition, side effects of HUC-MSC transplantation must be considered. Resolving these issues and obtaining a better understanding of the biology of HUC-MSCs, transplantation of these cells will certainly gain a broader application in the treatment of liver diseases.

#### Application of HUC-MSCs in systemic lupus erythematosus

Systemic lupus erythematosus (SLE) is an autoimmune inflammatory disease of the connective tissue involving multiple organs. SLE affects prevalently young women. In most patients, traditional therapies for SLE can manage the condition but are associated with a high rate of adverse reactions, such as infection, ovarian failure, malignant tumors, osteoporosis, and other diseases, seriously affecting the patient’s quality of life. The immunoregulatory function of HUC-MSCs had been widely employed for the treatment of various autoimmune diseases, particularly in cases of severe and refractory SLE that had failed to respond to pharmacologic therapy, and some beneficial effects have been obtained.

The treatment of SLE by HUC-MSCs is safe and effective [34]. The overall survival rate of patients treated with HUC-MSCs is more than 80%, the remission rate varied among different studies, the recurrence rate was approximately 20% [35, 36], and BILAG or SLEDAI score was significantly reduced. In addition, serum levels of albumin, antibodies, and the complement, as well as the number of peripheral blood leukocytes, platelets, and 24-h proteinuria level, were all improved [35, 36]. HUC-MSCs can play a role in the treatment of SLE by inhibiting the proliferation of T cells, increasing the number of Treg cells, inhibiting the expansion of Tfh cells, maintaining the balance between T helper 1 and T helper 2 cells (Th1/Th2), and decreasing the level of TNF-α and IL-17 [37, 38]. In addition, certain microRNAs (miRNAs) are implicated in immune diseases, and the treatment of SLE by HUC-MSCs upregulated the expression of miR-153-3p and miR-181a [39, 40]. All these effects should become the subject of future research.

The findings discussed above point to two possible mechanisms by which HUC-MSCs can treat SLE: (1) inhibition of the proliferation of T and Tfh cells, upregulation of Treg cells, maintaining the Th1/Th2 balance, and decreasing the level of TNF-α and IL-17 and (2) regulation of the expression of certain miRNAs. Several in vitro and in vivo studies have demonstrated the immunomodulatory properties of HUC-MSCs, providing basic science support for the application of these cells in clinical practice. Although the current clinical application of the research on HUC-MSCs begins to materialize and shows good prospects, the possibility of excessive immunosuppression by HUC-MSCs creates the risk for infection and tumorigenesis. The possibility of this type of adverse effects necessitates further research and in-depth discussion.

#### Application of HUC-MSCs in arthritis

Arthritis is an inflammatory disease affecting joints and surrounding tissues. Its etiology is complex and mainly related to an autoimmune reaction, infections, and trauma. Traditional treatments do not effectively solve the problem of the lack of immune tolerance mechanisms and are burdened by obvious side effects. The use of stem cells became a new therapeutic strategy for this disease. HUC-MSCs have been shown to effectively treat arthritis by differentiating into osteoblasts [41]; inhibiting the proliferation and promotion of apoptosis in T lymphocytes; reducing the secretion of IL-1, IL-6, IL-7, IL-17, and TNF-α; and suppressing the inflammatory response [42–44]. After treatment, the joint function and quality of life were significantly improved, as documented by the Lysholm score, WOMAC score, SF-36 scale score, health index (HAQ), and joint function index (DAS28) [45, 46].

HUC-MSCs also have a chondroprotective effect, which is considered to depend on the reduction of inflammation, which delays cartilage destruction. At the same time, HUC-MSCs inhibited the expression of MMP-13, collagen type X α1 chain, and cyclooxygenase-2, and enhanced the proliferation of chondrocytes, while osteoarthritis chondrocytes promoted HUC-MSCs to differentiate into chondrocytes [47, 48]. Additionally, HUC-MSCs have anti-fibrotic properties and may affect the course of arthritis by the secretion of HGF [6]. It has been documented that the treatment regimen consisting of a single injection of HUC-MSCs did not provide a satisfactory outcome, and in clinical practice, 3–5 rounds of administration of the cells are generally recommended [49].

In summary, HUC-MSCs can treat arthritis through the following mechanisms: (1) differentiation into cartilage or osteoblasts to repair the cartilage and regenerate the knee cartilage; (2) the release of soluble molecules such as cytokines, growth factors, and immunomodulatory factors to exert an immunomodulatory effect; and (3) inhibition of the proliferation of immature dendritic cells (DC) and natural killer (NK) cells, suppression of cytokine cytotoxicity, induction of macrophage differentiation from pro-inflammatory M1 phenotype to anti-inflammatory M2 phenotype, and secretion of IL-10 and nutritional factors. These properties of HUC-MSCs reduce inflammation and promote tissue repair. In addition, HUC-MSCs can inhibit the proliferation of T cells and B cells, and the immunomodulatory properties of HUC-MSCs significantly weaken the progress of osteoarthritis [44]. Therefore, HUC-MSCs have the potential for broad applications in the treatment of arthritis.

#### Application of HUC-MSCs in brain injury and cerebrovascular disease

The incidence of death and disability in cerebrovascular diseases such as brain injury and stroke is high. Traditional drug therapies do not provide satisfying results, and the sequelae of the damage can be severe, indicating that an effective treatment for stroke and other diseases is not available. It has been shown that the motor and nerve function scores in patients treated by HUC-MSC transplantation were improved [50], implying that this therapeutic modality can significantly reverse the brain function injury. Animal experiments demonstrated that HUC-MSC transplantation increased the release of VEGF, stimulated angiogenesis [51], and produced an anti-inflammatory effect by reducing the level of inflammatory factors [52]. Additionally, HUC-MSCs reduced neuronal apoptosis by increasing the expression of glial cell-derived neurotrophic factor (GDNF) and BDNF and reducing the number of hypertrophic microglia/macrophages, thus generating a neuroprotective effect [52, 53]. Intranasal administration of HUC-MSCs or HUC-MSC-ex to treat brain injury and cerebrovascular disease had also received a significant amount of attention in recent years as a noninvasive and safe treatment [54, 55]. HUC-MSC-ex can inhibit the expression of inflammation-related genes and pro-inflammatory factors. Moreover, the infusion of exosomes increases myelin formation and decreases glial hyperplasia. HUC-MSC-ex can regulate the activation of microglia and astrocytes, reduce the level of TNF-α and IL-1β, and increase the formation of IL-10, BDNF, and glial cell-derived neurotrophic factor [54–56]. These exosomes produce an anti-inflammatory effect and enhance nerve function. The methods for delivering HUC-MSCs for brain injury include lumbar puncture, arterial and venous infusion, direct injection into the brain, and implantation on biomaterials [50, 57]. The intravenous injection could lead to most HUC-MSCs being stranded in the lungs and failing to migrate to the brain or other organs. The arterial infusion provides a relatively broader distribution in the organism than intravenous infusion [57, 58]. The therapeutic effect of the combination of HUC-MSCs with other drugs or adjuvant therapy produces better outcomes than a single therapy. For example, HUC-MSC transplantation combined with minimally invasive hematoma aspiration for cerebral hemorrhage, or combined with nimodipine for radiation-induced brain injury, provided results indicating that the therapeutic effects were superior to those of a single therapy [59, 60].

HUC-MSCs and their exosomes treat cerebrovascular diseases primarily by inducing an anti-inflammatory effect through the downregulation of inflammation-related genes and reduction in the level of pro-inflammatory factors while promoting the release of VEGF and neovascularization. Moreover, HUC-MSCs and HUC-MSC-ex increase the level of BDNF and glial cell-derived neurotrophic factor, which protect neurons and enhance their function. Due to the biological characteristics of HUC-MSCs and the uniqueness of cerebrovascular diseases, different transplantation pathways can affect the number and spatial distribution of HUC-MSCs that home to the brain parenchyma, thereby affecting the therapeutic effect of these cells. Therefore, the route of delivery of HUC-MSCs for the treatment of cerebrovascular diseases has become the focus of current research.

#### Application of HUC-MSCs in cardiac diseases

Heart diseases are the major cause of mortality worldwide, with approximately 20 million people aged 30–70 years dying from the disease every year. At present, the disease tends to affect younger individuals. The available treatments include heart transplantation, surgical interventions, and pharmaceutical therapies. Surgical treatment is typically associated with complications and generally is not recommended unless the condition is severe. Even if the patients survive and the condition improves, a long-term maintenance treatment is necessary. HUC-MSCs offer a relatively safe and effective alternative therapy for heart diseases.

HUC-MSCs have been shown to treat and relieve various cardiovascular diseases, including myocardial infarction, heart failure, myocardial ischemia, and myocarditis. These cells promote cardiac tissue regeneration and angiogenesis, inhibit inflammation [61], and significantly reduce infarct size and mortality. Also, transplantation of HUC-MSCs improves the New York Heart Association functional class and the results of the Minnesota Living with Heart Failure Questionnaire and 6-min walk test, significantly improving patients’ quality of life [62, 63]. The mechanism of HUC-MSCs’ effects on the heart is not fully understood yet, but previous studies documented that HUC-MSCs can have an anti-apoptotic function by increasing the expression of anti-apoptotic protein Bcl-2 and decreasing the expression of pro-apoptotic proteins Bax and pro-caspase-9 [64]. The differentiation of HUC-MSCs into cardiogenic cells can be promoted by the overexpression of NK 2 homeobox 5 (Nkx 2.5) and pygopus family PHD finger 2 (PyGO2) proteins and the regulation of the p53-p21 pathway [65–67]. The induced cardiomyocytes can form intercalated discs with myocytes of the host cell, forming a functional syncytium and directly participating in the contraction of the heart. In this manner, the transplanted cells enhance the local contractile function of the myocardium, reduce the necrotic infracted area, and increase ejection fraction. The long-term follow-up found that the induction of blood vessel formation was also an essential part of heart repair after injury [63, 64]. Importantly, HUC-MSCs can secrete HGF to exert anti-inflammatory effects [62]. Interleukins, TNF-α, colony-stimulating factor, and chemotactic cytokines generated by HUC-MSCs can inhibit inflammation in the myocardium and reduce the degree of cardiac fibrosis. HUC-MSCs can affect the expression of the MMP/TIMP system in myocardial fibroblasts through the ERK1/2 pathway, inhibit the production of TGF-β that is related to myocyte hypertrophy, and contribute to the prevention of myocardial fibrosis [68].

HUC-MSCs can also upregulate the level of superoxide dismutase (SOD) and glutathione (GSH), reduce the concentration of malondialdehyde (MDA) in infarcted myocardium, and reduce oxidative stress and extracellular matrix (ECM) remodeling [64]. HUC-MSC can also indirectly play the role of treating heart diseases by regulating the expression of miRNA, lncRNA, and circRNA [10–12].

In summary, HUC-MSCs perform a therapeutic function in heart-related diseases by the following mechanisms: (1) differentiation into cardiomyocytes to improve heart function, (2) differentiation into vascular endothelial cells to promote angiogenesis and blood supply, (3) improvement of cardiac performance by inhibiting myocardial cell apoptosis, (4) anti-inflammatory and anti-fibrotic activity through paracrine effects, and (5) regulating the expression levels of miRNAs, lncRNAs, and circRNAs involved in cardiac repair. HUC-MSCs have broad prospects for clinical application in the treatment of cardiac diseases.

However, several issues related to the application of HUC-MSCs in cardiac therapies remain to be investigated, such as the timing, quantity, and administration mode of transplanted cells; the mobilization and homing of the cells; and the safety and long-term outcomes of cell transplantation. Therefore, in-depth studies of the specific mechanism underlying the therapeutic effects of HUC-MSCs for cardiovascular diseases are necessary to enable the future use of these cells to treat heart-related diseases and restore cardiac function.

#### Application of HUC-MSCs in spinal cord injury

Spinal cord injury (SCI) is a cross-sectional injury of the spinal cord caused by trauma, inflammation, and other factors. SCI results in impairment or loss of motor, sensory, and other nerve functions below the site of injury. Once SCI takes place, particularly in cases of traumatic injury, the patient should be rescued as soon as possible to maintain blood volume and prevent neurogenic shock. After resuscitation, the patient should be given drug therapy to repair damaged nerve fibers and maintain spinal cord stability to prevent further nerve injury. However, the effects of pharmaceuticals are very limited, and the adverse effects of corticosteroids are significant. Given this backdrop, HUC-MSCs, with their strong proliferation, differentiation, and self-renewal potential, attracted the interest of scientists. HUC-MSCs represent a new treatment strategy for spinal cord injury, which is effective and engenders few side effects. Thus, treatment with HUC-MSCs is expected to become an alternative therapy for SCI.

Clinical research showed that HUC-MSC treatment of patients with SCI could restore intestinal and bladder function and significantly improve sensation, movement, and self-care ability, as indicated by higher American Spinal Injury Association scores and daily life activity scores [69, 70]. Several studies have documented that timely transplantation of HUC-MSCs effectively treats SCI by promoting the recovery of nerve function. Repeated doses of HUC-MSCs alone or in combination with human neural stem cells (HNSCs), GDNF, and hypoxic conditions enhance the outcome of cell therapy [71–73]. The mechanisms by which HUC-MSCs ameliorate the effects of SCI include the inhibition of the mitogen-activated protein kinase (MAPK) pathway that is activated after SCI, and reduction in the apoptosis of spinal cord neurons [74]. HUC-MSCs also decreased the secretion of inflammatory cytokines IL-6, IL-7, and TNF-α, thereby reducing the inflammatory response at the site of injury [75, 76] and promoting neuronal regeneration and reducing the formation of glial scar [73]. However, detailed mechanisms and efficacy need to be established in large-scale clinical trials.

In summary, HUC-MSCs can treat SCI cord injury mostly by two mechanisms: (1) reduction of the apoptosis of spinal cord neurons and (2) suppression of the secretion of inflammatory cytokines IL-6, IL-7, and TNF-α secretion, resulting in the inhibition of the inflammatory response at the injured site, promotion of neuronal regeneration, and reduction in the formation of glial scars. Although rapid progress has been made in the use of HUC-MSCs for the treatment of SCI, their clinical application continues to face many problems. Future efforts should focus on the standardization of various HUC-MSC technologies, identification of the mechanisms of neural repair, a better understanding of signaling molecules and conduction pathways, and comprehensive assessment of different treatment protocols.

#### Application of HUC-MSCs in respiratory diseases

At present, there are not many instances of the use of HUC-MSCs to treat the respiratory system. Typical diseases include acute lung injury, bronchial asthma, and chronic obstructive pulmonary disease. For respiratory diseases, HUC-MSC treatment significantly elevated functional scores and increased patient survival [77]. Intrapulmonary infusion of HUC-MSCs can reduce lung inflammation and improve lung function through paracrine KGF. Additional mechanisms leading to the reduction of lung inflammation include the overexpression of interleukin-33 (IL-33) and antagonist interleukin-1 receptor-like-1 (IL-1 receptor-like-1), inhibition of protein extravasation, suppression of the proliferation of neutrophils, and secretion of inflammatory factors TNF-α, IL-6, and macrophage inflammation protein 2 (MIP-2) [78–81]. The beneficial effect of MSCs on asthma is achieved mostly by regulating immune responses and anti-inflammatory activity. Intrapulmonary injection of MSCs can reduce airway inflammation in asthma by adjusting the ratio between Th1, Th2, and Tregs cells, and attenuate airway hyperresponsiveness by inhibiting the Th17 signaling pathway [82]. The role of HUC-MSCs in chronic obstructive pneumonia relies mostly on the following mechanisms: (1) reduction of airway inflammation; inhibition of the secretion of inflammatory factors such as IL-1β, TNF-α, TGF-β, IL-6, and IL-8; inhibition of the oxidative stress caused by inflammation; and anti-apoptotic signaling; (2) promotion of the secretion of growth factors, such as KGF, stem cell growth factor, HGF, and EGF; activation of tissue repair; and enhancement of lung perfusion; (3) remodeling of pulmonary vasculature and improvement of lung function; and (4) regulation of lung function through the expression of miR-410, miR-451, and miR-145 [83–85].

HUC-MSCs have been used in the treatment of lung diseases for more than 10 years. HUC-MSCs protect lung tissue primarily through anti-inflammatory and anti-fibrotic activity, immune regulation, and paracrine mechanisms. HUC-MSCs also indirectly treat lung diseases by regulating the expression of microRNAs. In short, these cells provide an important therapeutic effect and have a wide range of applications in the treatment of respiratory diseases.

#### Application of HUC-MSCs in viral infections

A recent study reported the use of HUC-MSCs to treat the coronavirus disease-19 (COVID-19) [86]. Currently, more investigations are being carried out in more countries to study the efficacy and the underlying mechanism. HUC-MSCs were also effective in restoring impaired alveolar clearance and protein permeability in patients infected with the H5N1 virus [87]. HUC-MSCs improved immune reconstitution in immune non-responders (INRs) and may represent a new immunotherapy tool for reversing immunodeficiency in HIV-1-infected INRs [88]. In general, HUC-MSC treatment of viral diseases improves clinical symptoms by regulating the immune function of the patients. Importantly, HUC-MSCs are a safe and feasible source of human diploid cells (HDCs) for the production of antiviral vaccines [89].

Antiviral therapy utilizing HUC-MSCs has provided certain therapeutic effects, but this application of HUC-MSCs only begun to be studied. Understanding involved mechanisms or their combinations, adverse reactions, usage and dosage, and treatment methods is minimal. Clinical treatments should proceed with extreme caution, and scientific research needs urgently to be developed.

#### Application of HUC-MSCs in other diseases

HUC-MSCs reduced the incidence of the graft versus host disease (GVHD) and relieved its clinical symptoms by immunomodulatory effects. Specifically, HUC-MSCs increased the number of B lymphocytes, Treg cells, and the Th1/Th2 ratio and decreased the number of NK cells in patients affected by GVHD [90]. In autistic patients, HUC-MSC transplantation significantly increased the level of HGF, BDNF, and nerve growth factor (NGF) in cerebrospinal fluid [91]. In patients with femoral head necrosis, HUC-MSCs significantly reduced the necrotic volume of the femoral head and increased the oxygen release index. After intra-arterial delivery, HUC-MSCs migrated to the necrotic area of the bone and differentiated into osteoblasts, providing a therapeutic effect [92]. In patients with long-term infertility due to premature ovarian failure, HUC-MSC therapy preserved ovarian function by increasing estradiol concentration, improving follicular development, and increasing the number of sinus follicles [93]. In patients affected by inflammatory enteritis, treatment with HUC-MSCs allowed a significant reduction in corticosteroid dosage, which might be related to regulating the expression of IL-6, IL-7, and IL-10 [94]. In Alzheimer’s disease, HUC-MSCs ameliorated cognitive dysfunction and cleared the deposits of amyloid β, stimulated the activation of brain microglial cells, reduced the level of pro-inflammatory cytokines, increased anti-inflammatory cytokines, and suppressed neuroinflammation. Together, these changes had an important effect on the outcome of Alzheimer’s disease [95]. In multiple sclerosis patients, intravenous infusions of HUC-MSCs were safe and effective, and no active lesions were found on MRI scans of the brain and the cervical spinal cord after a follow-up of 1 year [96]. Thus, HUC-MSCs have provided satisfactory therapeutic benefits in several diseases, but the theoretical basis of these effects remains to be established.

### Basic research on HUC-MSCs related to clinical applications

#### Establishment of an HUC-MSC cell bank

Most of the HUC-MSCs currently used clinically are provided by stem cell banks that collect, prepare, and store stem cells on a large scale. Stem cell banks are also called “Life Banks.” The HUC-MSC cell bank is one of a wide variety of stem cell banks. At present, there are few dedicated HUC-MSC cell banks, and in most cases, HUC-MSCs are supplied by comprehensive stem cell banks. In 2004, the world’s first stem cell bank that stored HUC-MSCs was opened in the UK. The National Institute for Biological Standards and Control (NIBSC) was responsible for the operation and storage of embryos, fetuses, adult tissues, and various stem cell lines, including HUC-MSC lines. In April 2006, the first Chinese HUC-MSC cell bank was established in Tianjin, with a storage capacity of tens of thousands of copies. It has established standardized protocols of stem cell isolation, identification, culture, expansion, storage, supporting technologies, and quality control, and the cell bank developed the first domestic technical standard for HUC-MSCs and fulfilled the ISO 9001:2008 quality management system requirements [97]. In 2011, Dr. Khushnuma Cooper reported a method for building an HUC-MSC cell bank and believed that HUC-MSCs have a wide range of clinical applications [98].

An HUC-MSC cell bank could provide high-quality stem cell “seeds” from legal sources for the clinical application, and many companies and hospitals have the ability to build HUC-MSC cell banks. However, there are no uniform quality control standards in stem cell banks, which will directly affect the outcomes of clinical treatment. This issue represents the biggest problem of stem cell banks and should be resolved as soon as possible.

#### Tumorigenicity of HUC-MSCs

The tumorigenicity of transplanted HUC-MSCs, which will affect the therapeutic effect, is one of the current concerns. The experiments on tumorigenicity safety conducted by Jun-Won Yun and coworkers in mice did not detect tumors related to HUC-MSCs [33]. Also, cultured HUC-MSCs injected into rats did not form tumors [99]. Yong Wang and collaborators demonstrated that when HUC-MSCs fuse with esophageal cancer cells, they induce apoptosis and promote the benign phenotype of cancer cells [100]. HUC-MSCs stimulate the growth of ovarian tumors through cell-to-cell communication but can reduce tumorigenesis after fusion with ovarian cancer cells [101]. HUC-MSCs can exert an anti-tumor effect also by affecting transcriptional regulation in leukemia cells. Additionally, HUC-MSCs can enhance the proliferation and migration of cancer cells [102, 103]. So far, there is no conclusive proof of HUC-MSC tumorigenicity or their ability to promote the development of cancer already present in the organism. These questions can only be answered by expanding the number of samples and performing multi-center trials.

#### Therapeutic effects of different generations of HUC-MSCs

The therapeutic effects of HUC-MSCs are dependent on the number of their generations in culture, HUC-MSCs at less than 10 passages have better cardiogenic differentiation ability, while at passages 11–20, the differentiation towards nerve cells is evidently enhanced [104]. In addition, CD29, CD44, CD73, CD90, and CD105 are highly expressed at passages 1 to 16, and CD166 is highly expressed at passage 2. HUC-MSCs at passages 4 and 16 have a strong potential to differentiate into osteoblasts. Therefore, HUC-MSCs at late passages show stable bone differentiation capacity [105, 106]. In the treatment of acute liver failure, HUC-MSCs harvested at different passages show distinct effects. Cells at passage 5 are more potent than passage 10 cells in homing to the liver, as well as in enhancing proliferation and inhibiting apoptosis of liver cells [107]. These findings imply that cells at different passages may have distinct therapeutic effects in various diseases, and selection of an appropriate passage of HUC-MSCs according to the type of disease may be required to achieve optimal therapeutic results. However, no conclusive evidence for the relationship between the number of cell passages and disease treatment effects is currently available. To reach definitive conclusions, the sample size should be expanded, and the principles of evidence-based medicine should be followed.

## Conclusions

Currently, mesenchymal stem cells are a hot research subject in the field of regenerative medicine. This article analyzes five functions of mesenchymal stem cells relevant to clinical applications and their therapeutic effects in eleven types of diseases, including liver and respiratory system disorders.

Before 2006, the research of stem cells was mainly directed at the identification of biological characteristics of stem cells. In 2006, the International Association for Cell Therapy (ISCT) proposed a unified definition of mesenchymal stem cells, which became the identification standard used worldwide. Only cells that meet the following three criteria simultaneously can be classified as mesenchymal stem cells: (1) anchorage-dependent growth, (2) expression of certain specific antigens (markers) on the cell surface, and (3) ability to differentiate into adipocytes, osteoblasts, and chondrocytes. In subsequent years, great progress has been made in the use of HUC-MSCs for clinical application. An increasing number of relevant studies have been conducted, and the research methods have matured. The comparison of the therapeutic effects of HUC-MSCs with those of traditional treatments demonstrates that HUC-MSCs not only improve survival but also significantly ameliorate various clinical symptoms of the disease, markedly improving patients’ quality of life [30]. Since HUC-MSCs play a therapeutic role from many aspects, and the pathogenesis of each disease is also different, for a certain disease, HUC-MSCs may play a part of the function to achieve the therapeutic effect. However, how to maximize the function of HUC-MSCs required by the disease in a specific disease is an important topic of current research. Meanwhile, the effects of the treatment are not always persistent, the mechanistic underpinnings of the beneficial effects are not fully understood, and large-scale production is at its infancy. These factors negatively affect the clinical use of HUC-MSCs. Despite these limitations, in suitable clinical indications, mesenchymal stem cells can still provide therapeutic benefits. Throughout many past clinical trials, the unclear results were mostly caused by inappropriate clinical endpoints, inadequate design of the trial, unclear mechanism of action, and—as a major factor—unstable cell quality. In addition, technical issues such as cell transplantation dose, delivery route, choice of the time window for cell transplantation, injection rate, and transplantation frequency remain to be solved to ensure the optimal clinical outcome. Therefore, future development of HUC-MSCs should address the following problems: (1) the necessity to design large-scale, multi-sample, multi-center, long-term follow-up studies to verify the efficacy and safety of HUC-MSC in the treatment of various clinical diseases; (2) the establishment of a stem cell bank and reinforcement of the development and quality control of stem cell preparations; and (3) intensification of research on HUC-MSCs, mechanisms of action, tumorigenicity, and safety. At present, the understanding of their function and underlying mechanisms is rather limited.

HUC-MSCs bring new hope to the future of regenerative medicine. Further in-depth research and clinical applications of mesenchymal stem cells will certainly benefit mankind in the near future. Mesenchymal stem cells have a remarkable potential to differentiate, strong proliferation capacity, and low immunogenicity. Their use is not limited by moral and ethical restrictions, and they are easy to prepare on an industrial scale. They may become pluripotent stem cells with the best prospects for broad clinical applications.

## Supplementary Information

**Additional file 1: Table S1.** Application of HUC-MSCs in clinical treatment.

## Data Availability

Not applicable.

## Abbreviations

ALT: Alanine transaminase
AST: Aspartate aminotransferase
BDNF: Brain-derived neurotrophic factor
circRNA: Circular RNA
COPD: Chronic obstructive pulmonary disease
COVID-19: Coronavirus disease-19
DC: Dendritic cells
ECM: Extracellular matrix
GDNF: Glial cell-derived neurotrophic factor
GSH: Glutathione
GVHD: Graft versus host disease
HUC-MSCs: Human umbilical cord mesenchymal stem cells
HNSCs: Human neural stem cells
HDCs: Human diploid cells
HGF: Hepatocyte growth factor
IL-1: Interleukin-1
IL-4: Interleukin-4
IL-6: Interleukin-6
IL-7: Interleukin-7
IL-8: Interleukin-8
IL-10: Interleukin-10
IL-17: Interleukin-17
IL-33: Interleukin-33
IL-1β: Interleukin-1β
INRs: Immune non-responders
lncRNA: Long non-coding RNA
MIAT: Myocardial infarction-related transcript
MMP: Matrix metalloproteinases
MDA: Malondialdehyde
miRNA: MicroRNA
MAPK: Mitogen-activated protein kinase
NK: Natural killer cell
NGF: Nerve growth factor gene
NSCs: Neural stem cells
Nkx 2.5: NK 2 homeobox 5
ODI: Oxygen release index
PyGO2: Pygopus family PHD finger 2
SLE: Systemic lupus erythematosus
SOD: Superoxide dismutase
SCI: Spinal cord injury
T1DM: Type 1 insulin-dependent diabetes mellitus
T2DM: Type 2 insulin-independent diabetes mellitus
TNF-α: Tumor necrosis factor-α
Th1/Th2: T helper 1/T helper 2
TGF-β: Transforming growth factor β
VEGF: Vascular endothelial growth factor
