# Supplementary material for: What is the impact of human umbilical cord mesenchymal stem cell transplantation on clinical treatment?
Source: Stem Cell Res Ther. 2020 Dec 1;11:519. doi: 10.1186/s13287-020-02011-z (PMC7705855; doi:10.1186/s13287-020-02011-z)
Supplement: Supplementary file 1 — Additional file 1: Table S1. Application of HUC-MSCs in clinical treatment. [file 13287_2020_2011_MOESM1_ESM.docx]

| Tab. S1 Application of HUC-MSCs in clinical treatment | | | |
| --- | --- | --- | --- |
| Authors | Diseases | Type of study | Brief mechanism |
| Yaqi Yin, Xiaoya Sun, Kong D | type 2 diabetic | pre-clinical | Enrichment to pancreatic islet, and differentiate into functional islet cells; macrophage polarization, blocking the activation of NLRP3 inflammasome |
| Jinquan Cai, Kong D | Diabetes | clinical | insulin, C peptide and Treg cells↑; glycosylated hemoglobin fasting glucose and daily insulin requirement↓ |
| Ying Wang, Ning Zhou, Qing-Song Zhao | Diabetic Foot | pre-clinical | Targeting ulcer tissues and differentiation into functional islet-like cells cytokeratin 19, VEGF, BDNF↑ promote the epithelialization of ulcerated tissue |
| Treiman D M | Diabetic Foot | clinical | improvement in ankle-brachial pressure index, transcutaneous oxygen tension, and claudication distance; newly formed vessels increased, and ulcers healed partially or completely |
| Lian Chen, Yuxin Xian | Diabetic Nephropathy | pre-clinical | anti-apoptotic proteins↑; blood glucose, blood urea nitrogen, and 24-hour urinary albumin excretion rate↓ |
| Shichang Zhang, Chun Yue | diabetic wounds | pre-clinical | macrophage polarization; IL-10, VEGF↑ |
| Seyedeh-Sara Hashemi PhD | diabetic wounds | clinical | The healing time and the size of the wound significantly shorten |
| Zhang Wei, Chuan Yu | diabetic retinopathy | pre-clinical | retinal microvascular permeability and vessel leakage↓; BDNF，Thy-1，IL - 1β and IL - 6 mRNA↓ |
| Guo-Zun Zhang | liver fibrosis/cirrhosis | pre-clinical | MMP-13↑, TIMP-1↓; accelerated the degradation of fiber matrix and promoted the apoptosis of HSCs; differentiated into functional hepatocytes |
| Su Yeon An | Liver Fibrosis | pre-clinical | fibrotic factors, collagens, metalloproteinases, TGFβ, and Smad proteins in the TGFβ signaling pathways↓;  HSCs proliferation and collagen production↓ |
| Linrui Jiang | acute liver failure | pre-clinical | NLRP3 inflammasome, ALT, AST↓, play an anti-inflammatory role |
| Jia Yao | hepatic ischemia-reperfusion injury | pre-clinical | infiltration of neutrophils, and oxidative stress and apoptosis of liver cells↓ |
| Fei Gu | Lupus Nephritis | clinical | the overall rate of survival was 95 %; BILAG scores, SLEDAI scores ↓ |
| Dandan Wang | Drug-Resistant Systemic Lupus Erythematosus | clinical | the overall rate of survival was 84 %; SLE Disease Activity Index scores, serum albumin, complement C3, peripheral white blood cell, platelet numbers, and proteinuria levels, continued to improve |
| Dandan Wang | systemic lupus erythematosus | clinical | Treg cells, Tfh cells, TNF-α, IL-17↓;  maintaining the balance between Th1and Th2 |
| Dan Li | systemic lupus erythematosus | pre-clinical | upregulated the expression of miR-153-3p |
| B Zheng | systemic lupus erythematosus | clinical | upregulated the expression of miR-181a |
| Bei-Ying Zhang, Jose Matas | Arthritis | pre-clinical | Th17 subset, Tfh cells, IL-1β, IL- 6, IL- 7, IL-17, TNF-α↓, and reduce the inflammatory response; Treg cells↑ |
| Liming Wang | Rheumatoid Arthritis | clinical | WOMAC score, joint function index (DAS28), C-reactive protein and Rheumatoid Factor↓ |
| Yali Wang | degenerative knee osteoarthritis | clinical | Lysholm score, WOMAC score and SF-36 scale score significantly improved |
| Hao Wang | Osteoarthritis | pre-clinical | MMP‑13, collagen type X α1 chain and cyclooxygenase‑2↓; proliferation of chondrocytes↑ |
| Jose Matas | Knee Osteoarthritis | clinical | repeated UC-MSC superior to active comparator |
| Dan Ma | rheumatoid arthritis | pre-clinical | inhibiting the proliferation and promoted apoptosis in T lymphocytes;  Th17 cell ratio, IL-17↓,Treg cell ratio, TGF-β↑ |
| Sen Wang | sequelae of traumatic brain injury | clinical | significant improvement in Fugl-Meyer Assessments (FMA) and Functional Independence Measures (FIM) |
| Hua-Jiang Dong, Lifeng Qi | Traumatic Brain Injury | pre-clinical | VEGF, GDNF , BDNF↑and stimulated angiogenesis; inflammatory factors↓ |
| Willie Lin | Cerebral Ischemia | pre-clinical | hypertrophic microglia/macrophages↓, newborn neurons↑ |
| Gierin Thomi, Byron Oppliger | Perinatal Brain Injury | pre-clinical | neuron-specific cell death, glial hyperplasia↓; normal myelination, neuronal cell counts↑; facilitates the maturation of oligodendrocyte lineage cells. |
| Sheng-Jie Shiue | Nerve Injury-Induced Pain | pre-clinical | TNF-α, IL-1β↓, IL - 10, GDNF , BDNF↑ |
| Yongjun Jiang | Stroke | clinical | Intra-artery infusion exhibits greater biological distribution compared to intravenous delivery |
| Qinghua Zhang | intracerebral hemorrhage | pre-clinical | HUC-MSCs transplantation combined with minimally invasive hematoma aspiration for cerebral hemorrhage |
| Gui-Hua Wang | Radiation-Induced Brain Injury | pre-clinical | HUC-MSCs transplantation combined with Nimodipine for radiation-induced brain injury |
| Lim Meikuang | Acute Myocardial Infarction | pre-clinical | TNF, IL-6↓, VEGF, platelet/endothelial cell adhesion molecule 1, connexin 43↑; Enhance angiogenesis and inhibit inflammation |
| Jorge Bartolucci | Cardiopathy | clinical | HGF↑; improved the New York Heart Association functional class and the results of the Minnesota Living with Heart Failure Questionnaire |
| Zhihua Fang | Heart Failure | clinical | improved six-minute walk test and left ventricular ejection fraction |
| Jing Ni | Myocardial Infarction | pre-clinical | angiogenesis, anti-apoptotic protein Bcl-2↑; extracellular matrix (ECM) remodeling, collagen deposition, pro-apoptotic proteins Bax and pro-caspase-9↓ |
| Changyi Zhang | dilated cardiomyopathy | pre-clinical | inhibited TNF‑α and the TGF‑β1/ERK1/2 fibrosis pathways |
| Xin-Long Wang | acute myocardial infarction | pre-clinical | Smad7 expression↑ |
| Bao Zhu | Aging-Induced Cardiac Dysfunction | pre-clinical | lncRNA metastasis-associated lung adenocarcinoma transcript 1↑ |
| Zhifeng Xiao, Wu-Sheng Deng | Acute Complete Spinal Cord Injury | clinical | restored intestinal and bladder function; sensation, movement, self-care ability, American Spinal Injury Association scores and daily life activity scores↑ |
| Lei Sun, C H Yang, Genlong Jiao | Spinal Cord Injury | pre-clinical | Repeated doses of HUC-MSCs or in combination with human neural stem cells (HNSCs), GDNF, and hypoxic conditions enhance the outcome |
| Guodong Sun | Spinal Cord Injury | pre-clinical | TNF-α, MIP-1α, IL-6 and IFN-γ↓ |
| Wu Ling-Ling | Spinal Cord Injury | pre-clinical | IL-6 and TNF-α↓, GDNF↑ |
| D-Z Tian | Spinal Cord Injury | pre-clinical | inhibition of thep38 MAPK pathway and reduction in the apoptosis of spinal cord neurons |
| Wei-wei Liu | paraquat-induced lung injury | clinical | significantly elevated functional scores and increased patient survival |
| Daming Liu | Radiation-Induced Lung Injuries | pre-clinical | chemokines and inflammatory cytokines↓, anti-inflammatory cytokines↑; Reduce inflammation and inhibit fibrosis |
| Zhi Wei Huang | Acute Lung Injury | pre-clinical | TNF-α, TGF-β₁, and IL-6↓, IL-10↑; Improved systemic inflammatory response and alveolar permeability |
| Yuhui Hao | Radiation-Induced Lung Injury | pre-clinical | reduce oxidative stress, inflammatory reactions, and TGF-β-Smad2/3 pathway activation |
| Hua Zhu | Acute Lung Injury | pre-clinical | paracrine secretion prostaglandin-E2, IL-6 and IL-13 |
| Louisa J Mathias | allergic asthma | pre-clinical | reduced airway inflammation and attenuate airway hyperresponsiveness |
| Liyang Dong | lung adenocarcinoma | pre-clinical | promote lung adenocarcinoma growth by transferring miR-410 |
| Ji-Song Liu | Burn-Induced Acute Lung Injury | pre-clinical | Regulation of the expression of miR-451 |
| Hayley Loy | Attenuating Influenza A(H5N1) Virus | pre-clinical | effective in restoring impaired alveolar fluid clearance and protein permeability |
| Zheng Zhang | HIV-1 | clinical | central memory CD4 T-cell counts, IFN-γ and IL-2↑; Effectively improve immune remodeling in INRs |
| Ping Chen | the Production of Human Viral Vaccines | pre-clinical | a source of human diploid cells (HDCs) for the production of antiviral vaccines |
| Lei Gao | Chronic Graft-Versus-Host Disease | clinical | Reduced the incidence and alleviated clinical symptoms; memory B lymphocytes, Th 1/Th2↑ |
| Q Li | Autism | clinical | HGF, BDNF and NGF↑ |
| Chun Chen | osteonecrosis | clinical | Differentiated into osteoblasts |
| Lijun Ding | Premature ovarian failure | pre-clinical | estradiol concentrations, antral follicles↑ |
| Fei Mao | Inflammatory Bowel Disease | pre-clinical | TNF-α、,IL-1β、IL-6、IL-7↓, IL-10↑ |
| Mao Ding | Alzheimer's Disease | pre-clinical | ameliorated cognitive dysfunction, stimulated the activation of brain microglial cells and modulated the levels of inflammatory cytokines; amyloid-β peptide↓ |

Tab. S1 The major clinical treatment and mechanism research of HUC-MSCs.
